# Effect of UV Radiation Exposure and Simulated Particle Erosion Damage on the Mechanical Behavior of Carbon/Glass Hybrid Composites

**DOI:** 10.3390/polym17070861

**Published:** 2025-03-24

**Authors:** Marcello de Vasconcelos Porto Hermanny Tostes, José Roberto Moraes d’Almeida

**Affiliations:** Chemical and Materials Engineering Department, Pontifical Catholic University of Rio de Janeiro, Rio de Janeiro 22451-900, Brazil; marcellotostes1@hotmail.com

**Keywords:** hybrid composite, UV aging, mechanical behavior, particle abrasion

## Abstract

The environments found in space research pose numerous challenges to the materials used in aerospace structures, such as high incidence of ultraviolet radiation (UV) and micrometeorite impacts. Therefore, this work analyzes the combined effects of exposure to UV radiation and damage caused by sandblasting on the mechanical performance of a hybrid composite of epoxy matrix reinforced with carbon and glass fibers to simulate service conditions both in low Earth orbit (LEO) and in exoplanet environments. The blasting was carried out with silica particles with dimensions compatible with those found in the dust of the Martian atmosphere, and the damage produced by these particles has dimensions similar to those observed in several impact/wear events of structures exposed to LEO conditions. A qualitative analysis of the effect of UV radiation carried out by colorimetry showed a significant change in the color of the material, which became more greenish and yellowish. This color change is indicative of degradation processes in the polymer matrix. FT-IR analysis showed an increase in the carbonyl band with increasing aging time, which is consistent with the color change measured in the material. However, the interlaminar shear strength was not affected by UV radiation in the time used in this work. This behavior was attributed to the fact that UV radiation initially causes deterioration only on the surface of the material. From the results of the bending tests, both the three-point bending test and impulse excitation test, it was found that the effect of UV radiation on the elastic modulus of the composites was more important than the effect of blasting damage. It was also observed that initial UV exposure, prior to sandblasting, has a synergistic effect on the deterioration of flexural strength.

## 1. Introduction

Fiber-reinforced polymer composites have numerous applications in various industrial areas, from repairs for the oil and gas industry [1] to structural components of satellites and aircraft [2,3,4,5]. In space exploration, composites are, in fact, materials with great prospects for increased employment due to their great design versatility and also due to their various recurring advantages, widely discussed in the literature, such as low density [3,6]. The design flexibility characteristics of composites have led to the development of aerospace structures capable of delivering the best performance, adaptability, safety, and functionality possible. Recent examples of the use of composites in space applications include structural supercapacitors [7] and various structural components of reentry vehicles [8]. The environments found in space research pose numerous challenges to the various materials used in aerospace structures. For example, in the low Earth orbit region, satellites are exposed to high incidences of ultraviolet radiation (UV) and micrometeorite impacts [4,9,10,11]. Temperature variations and the presence of atomic oxygen can also affect the performance of materials and/or devices in a satellite [4,9,12]. In the environment of nearby celestial bodies, such as Mars, exposure to UV and also continuous exposure to winds and dust can cause deterioration of the properties of materials used in the structures of the various equipment being placed on the planet’s surface, as well as in future permanent installations. Indeed, prolonged exposure to UV radiation on the surface of Mars can cause structural changes even in rocks or minerals [13,14], and sandstorms with winds of up to 20 m/s have been recorded in the vicinity of the Perseverance rover [15].

UV radiation is known to be a factor that causes high degradation and, consequently, compromises the properties of polymers [12,16] and polymer composites [17,18]. The energy of UV radiation that reaches the Earth’s surface varies between 290 and 460 kJ/mol, equivalent to the energy required for the dissociation of covalent bonds in polymer molecules [19]. Consequently, there may be chain scission, formation of double bonds, or unwanted cross-links [16]. Another possible effect of UV degradation is the formation of microcracks on the surface of the material, contributing to lower mechanical resistance and a non-uniform distribution of thermal stresses in the thickness of the material [20,21].

Although the isolated effects of UV exposure and dust on the behavior of polymers and polymer composites are known, their combined effect is less studied. Thus, this work aims to analyze, as a novelty approach, the combined effects of exposure to UV radiation and damage caused by sandblasting on the mechanical performance of a hybrid composite of epoxy matrix reinforced with carbon and glass fibers in order to simulate service conditions both in low Earth orbit and in exoplanet environments.

## 2. Materials and Experimental Methods

### 2.1. Material

The material used in this work was a hybrid composite of epoxy matrix reinforced with carbon and glass fibers. The glass fiber layers are nominally arranged at ±45°, while the carbon fiber layers are placed in a 0/90° configuration. This laminate configuration was manufactured to be symmetrical, with the fiberglass layers placed outside, and also to simulate a quasi-isotropic configuration. Thus, the coupling matrix ([B]_ij_) is zero, and the coupling terms of the stretching ([A]_ij_) and bending ([D]_ij_) matrices are, ideally, zero [22]. The laminate was manufactured using a mixed compression molding/manual lay-up technique. The fiber layers were manually placed inside the mold cavity, and the resin was poured and spread with a roller. After lamination, and to control the thickness of the manufactured laminate, the mold, coated with high-density polyethylene that served as a mold release agent, was closed and placed in a hydraulic press under a pressure of 4 tons, being kept under this pressure for 24 h. The nominal fiber ratio in the composite was 30% carbon fibers/70% glass fibers.

From the composite plates received, flexural test specimens (length: L = 110 mm, width: w = 25.8 mm and thickness: d = 5.3 mm) and interlaminar shear (ILSS) test specimens (length: L = 40 mm, width: w = 12 mm and thickness: d = 5.3 mm) were machined.

The microstructure of the material was analyzed by optical microscopy using the standard sample preparation procedure. That is, a cross-sectional sample of the composite was embedded, sanded using 200, 400, 600, and 800 mesh sandpaper, and then polished with alumina (0.3 μm). A Zeiss AxioCAM ICC-1 (Carl Zeiss, Jena, Germany) optical microscope was used for microstructure analysis.

### 2.2. Aging, Damage Introduction and Characterization Methods

The flexural and ILSS test specimens were subjected to UV aging in two chambers using a lamp with a wavelength of 234 nm and a power of 8 W. The samples were exposed on only one side to simulate a real application situation for periods of 60, 120, and 180 days. The effect of UV exposure was analyzed by FT-IR in the mid-infrared range (400 and 4000 cm^−1^), with a resolution of 4 cm^−1^ and 32 scans. The samples were analyzed using a Bruker Alpha II spectrometer (Bruker Corporation, Billerica, MA, USA) using the Attenuated total reflectance (ATR) technique.

A qualitative analysis of the effect of UV was also performed by colorimetry. In the context of composite aging, analyzing changes in color due to exposure to different environmental conditions can contextualize changes in the physical and mechanical properties of the material [23]. Color readings were performed on both the surface and the side of the test specimens. The colorimeter used was the Delta Color 450 G (Delta Color, São Leopoldo, Brazil), with an analysis field opening of 4 mm, standard illuminant D65, and standard observer at 10°. Six measurements were taken at each analysis point (surface or side) for each specimen. The CIELAB system was used, which considers the colorimetric coordinates L*, a*, and b*; where L* is the luminosity, a* and b* represent, respectively, the spectrum between shades of red to green and yellow to blue. The color difference was quantified by the parameter ΔE*, which summarizes the difference between all the coordinates of the CIELAB system, namely [23]:

(1) $${\Delta E}^{*}={\left[{\left({\Delta L}^{*}\right)}^{2}+{\left({\Delta a}^{*}\right)}^{2}+{\left({\Delta b}^{*}\right)}^{2}\right]\;}^{1/2}$$

Due to the variation in color perception by the human eye, some intervals can be adopted to characterize the degree of color change; thus, if ΔE* < 1, the color change is not perceptible to human vision. For values between 1 < ΔE* < 3.3, only trained people can perceive it, and it is not noticed by the general public. For values of ΔE* > 3.3, the change becomes perceptible to the naked eye for any individual [24].

Flexural test specimens were sandblasted on the surface exposed to UV to simulate the surface damage process of composites by external factors, such as erosion and abrasion due to exposure of the composite to a continuous flow of wind with sand. The blasting was carried out in the mid-section of the length of the flexural specimen. The specimens were blasted using the Peenmatic Micro 750S (Iepco Ag, Leuggern, Switzerland) equipment using silicon dioxide as the blasting agent for 2 min, under an outtake pressure of 3.2 bar. This time was chosen experimentally to simulate surface abrasion/erosion and generate damage similar to that reported by micrometeorite impacts [25] or due to continued abrasion [26,27]. The particle size of silicon dioxide was determined using the Malvern Morphology 4-particle analyzer (Malvern Panalytical Ltd, Malvern, UK). The particles were pulverized and dispersed on a slide and were analyzed to obtain their size distribution. The number of particles analyzed was 86,183.

The damage caused by sandblasting was assessed using the technique of reconstruction of individual images obtained by a motorized optical microscope (AxioImager.M2m, (Carl Zeiss, Jena, Germany)), which has controlled movement with respect to the x–y–z axes. Images were captured at several *z*-axis positions, but since optical microscopes have a low depth of focus, focus is only obtained at specific points, not for the entire surface. However, by controlling the *z*-axis movement, it is possible to obtain in-focus images of these small portions at various *z*-axis positions. A 3D representation of the surface topography is then obtained by assembling several individual images using image analysis techniques. Therefore, an image gathering all in-focus regions is obtained. A detailed explanation of the method is described elsewhere [28]. This image acquisition procedure was performed on 10 samples and was carried out using a lens with 5× magnification.

From the topographic image obtained, where the shades of gray represent height differences on the analyzed surface, roughness parameters were calculated in accordance with ISO 21920-2:2021 [29]. The parameters determined in this work were: (i) Rv, which represents the depth of the deepest valley (difference between the average height of the surface and the deepest valley); (ii) Rp, which represents the height of the highest peak (difference between the height of the highest peak and the average height of the surface); (iii) Rt, which represents the difference in total height of the texture, indicating the value of the maximum depth caused by blasting. These are amplitude parameters, which are related to the extreme characteristics of the profile [30,31].

The combined effect of UV exposure and the presence of damage caused by sandblasting, as well as the influence caused by exposure time, was analyzed by combining these effects, as shown in Table 1.

### 2.3. Mechanical Tests

Mechanical bending and interlaminar shear (ILSS) tests were performed on the composites as manufactured and after aging. Both tests were performed on an MTS 810 equipment (MTS Systems, Eden Prairie, MN, USA) with a 5 kN load cell. The 3-point bending tests were performed according to ASTM D790 standard [32] at a test speed of 3 mm/min. The span (L) used was 86 mm, so the L/d ratio was equal to 16, as specified in the standard. Ten specimens were tested per experimental condition. ILSS tests were performed according to ASTM D2344 [33] on the as-manufactured composites and on those subjected only to UV aging; due to the ILSS specimen’s dimension, locating the blast damage was impossible. Fifteen samples were tested per experimental condition at a loading rate of 1 mm/min, using a span of 30 mm.

### 2.4. Non-Destructive Test

**Figure 1:**
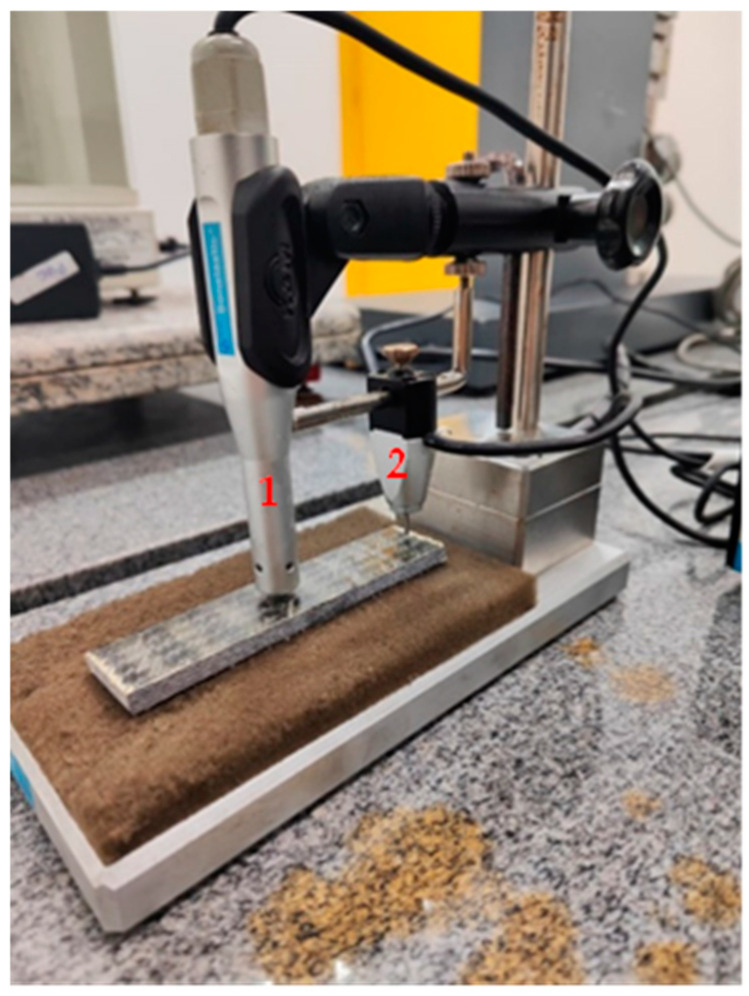
Experimental setup of the impulse excitation test, highlighting the positioning of the microphone (1) and the actuator (2) on the test specimen.

Before the destructive mechanical tests, the flexural specimens were analyzed using the non-destructive impulse excitation technique to analyze possible variations in the modulus of elasticity and damping factor due to aging/introduction of damage. The sonic test was performed using an electret microphone with a detection range from 0.5 to 20 kHz and an acquisition time of 0.6 s. A light actuator strikes the specimen surface every 30 s, and the generated signal is then processed by means of dedicated software (Sonelastic® (Sonelastic, Ribeirão Preto, Brazil)). Both the Young’s modulus and the damping factor are calculated automatically, following the procedures described by ASTM E 1876 [34]. The results reported are the average of 40 measurements. The test setup used is shown in Figure 1, where the relative position between the actuator and the microphone corresponds to the flexural-torsional boundary condition [35].

## 3. Results and Discussion

### 3.1. Characterization of Silica Particles

The analysis of the size of the silica used in the sandblasting/surface erosion process showed an average equivalent diameter of 2.39 μm, with particles ranging from 0.91 μm to 224.69 μm, with 90% of the particles having less than 3.13 μm in equivalent diameter. Although several studies report a wide range of values for the size of dust particles in the Martian atmosphere [36,37,38], the result obtained is consistent with the size of dust particles in the Martian environment. Values between <1 μm to 50–100 μm have been reported as present in the Martian atmosphere [39,40]. But are the smaller particles, ranging from approximately 1–5 µm in diameter, that can remain suspended for a long time [37,40]. It is, therefore expected that these particles are the ones that can cause the most damage to materials on the planet’s surface.

### 3.2. Microstructural Characterization of the Composite

**Figure 2:**
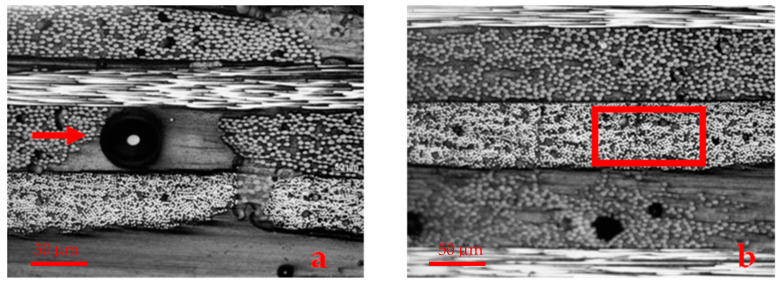
Representative aspect of the composite microstructure. It is possible to observe (**a**) pores in the matrix-rich region (→) and (**b**) defects due to lack of impregnation in the fiber layers (☐).

Figure 2 shows a representative region of the microstructure of the composite used in this work. It is possible to observe the presence of pores in the matrix-rich regions (Figure 2a), as well as voids due to lack of impregnation in the fiber layers (Figure 2b).

### 3.3. Characterization of Surface Damage Caused by Sandblasting

**Figure 3:**
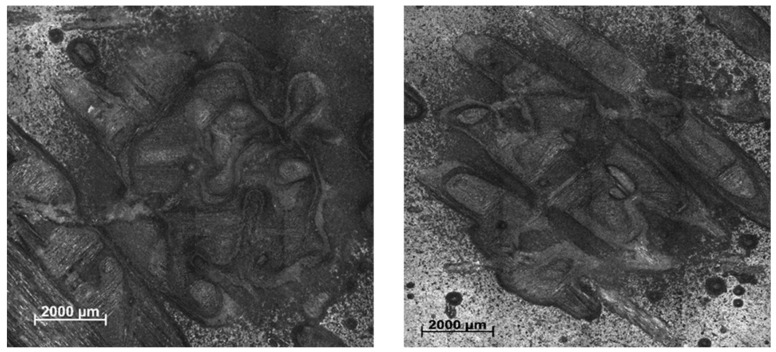
Example of damage caused by sandblasting. The damage area has no defined shape and there is ejection of material from the surface of the samples.

The surface of the sandblasted samples was evaluated to determine the type and depth of damage caused by sandblasting. Figure 3 shows, as an example, two sandblasted surfaces. It can be seen that the damage caused does not have a defined shape, and the sandblasted area has different depths. This type of defect, where an indentation and the ejection of the material around it are observed, is characteristic of low-energy impacts, such as those simulated in this work [41].

The parameters that characterize the topography of the sandblasted surfaces, obtained by reconstructing the images, are listed in Table 2. The Pt value, which indicates the average wear depth, is in agreement with the values reported in the literature. For example, tests simulating the effects of LEO report the depth caused by micrometeorites as 2.5 mm in CRFPs [42], while impactors with circular surfaces can cause defects with depths ranging from 0.100 to 0.499 mm, depending on the impact velocity [43] or from 0.12 to 1.32 mm depending on the fiber array [44].

### 3.4. Colorimetry

Table 3 shows the results of the colorimetric analysis of the composites before and after aging. The values of the variations in the color parameters, as well as the parameter ΔE* (Equation (1)), which defines whether the color change is noticeable or not, are listed in Table 4.

**Figure 4:**
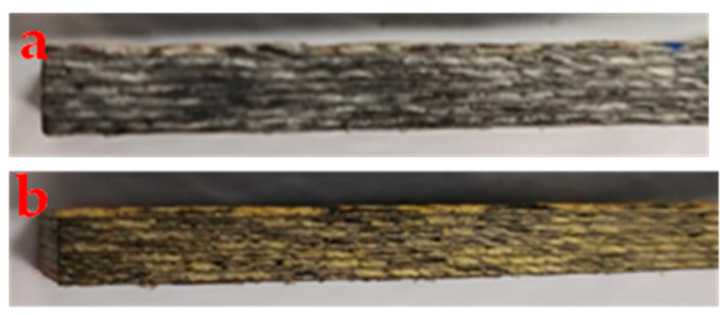
Side surface of the composite: (**a**) without aging; (**b**) aged.

From the ΔE* results, it can be seen that the color change is noticeable to the naked eye for all aging times since ΔE* > 3.3. Furthermore, since the difference in luminosity (∆L*) was positive, this indicates that the aged samples are becoming lighter than the as-manufactured samples. The variations of ∆a* and ∆b* indicate, respectively, that the samples are becoming greener and more yellowish [45]. An example of the color change is shown in Figure 4. Yellowing is characteristic of photo-oxidation processes caused by UV radiation [18,46].

### 3.5. Infrared Analysis (FTIR)

**Figure 5:**
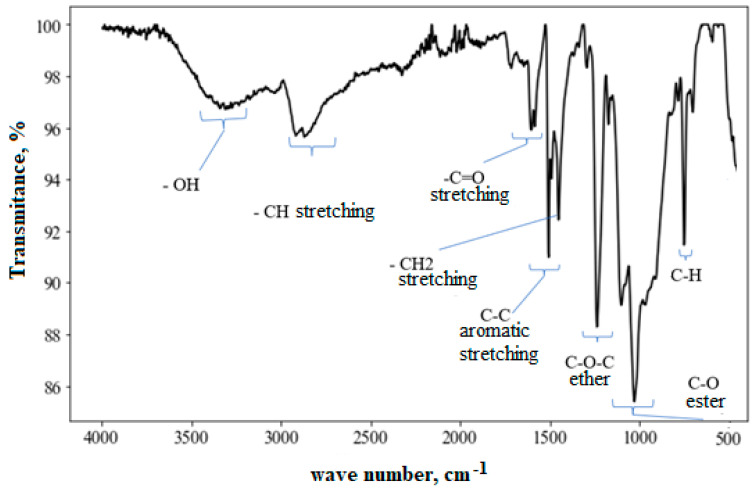
FT-IR spectrum of the composite as manufactured, highlighting the main absorption bands.

The FTIR spectrum of the as-fabricated composite is shown in Figure 5 and is in agreement with the spectra of tetraglycidyldiamino-diphenylmethane (TGDDM) epoxy resins reported in the literature [47]. One can observe the bands corresponding to C-C bonds between 1500–1450 cm^−1^ and C-H bonds between 800–700 cm^−1^, corresponding to aromatic rings [48]. In addition, between 2850–3000 cm^−1^, the presence of the C-H bond (of aliphatic hydrocarbon) is observed, in the range of 1750–1680 cm^−1^ the C=O bond (carbonyl), between 1300–1200 cm^−1^ C-O-C bonds (ethers) and in the range of 1100–1000 cm^−1^ C-O bonds (esters). It is also possible to notice the presence of -OH in the range of 3400–3200 cm^−1^ due to the absorption of moisture from the environment or from the hydroxyl groups present in the epoxy resin molecule.

The spectra of samples aged for up to 180 days did not show the appearance of new bands. However, there was a change in the relative height of the carbonyl band, which is a possible consequence of the effect of UV aging [46,49], which agrees with the color change determined by colorimetry (Figure 4). For the unaged material, the ratio between the carbonyl band and the C-C bond of the aromatic ring was 0.44, increasing to 0.68 to 0.75 for aging times of 60 to 120 days and reaching 1.14 for aging after 180 days.

### 3.6. Mechanical Behavior

#### 3.6.1. ILSS

**Figure 6:**
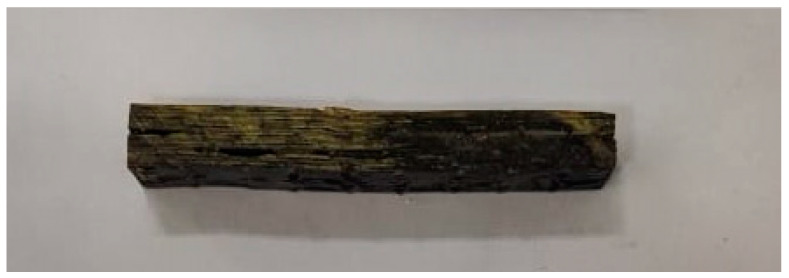
Sample aged for 60 days. Failure mode common to all aging times, classified as inelastic deformation.

The results of interlaminar shear strength are shown in Table 5. The samples aged for 60 days show a statistically significant reduction in relation to the material without aging (*p*-value = 0.009) and also in relation to the composites aged for 120 (*p*-value = 0.008) and 180 days (*p*-value = 0.021). On the other hand, the values of the samples without aging and aged for 120 and 180 days are statistically equal (*p*-value > 0.05). As previously discussed, UV radiation initially causes deterioration on the surface of the material [20,21,50]. Therefore, any increase in shear strength with increasing exposure time would not be expected but rather a continuous decrease or an initial decrease, followed by stabilization. The result obtained for the material aged for 60 days can be attributed to the lack of microstructural homogeneity of the composite, as highlighted in Figure 2. In fact, considering the values obtained for 120 and 180 days in relation to the material as manufactured, it can be stated that there was no effect of UV radiation on the interlaminar shear strength at the analysis time used in this work. The failure mode presented by the specimens corroborates this approach because, both before and after aging, it was of the inelastic deformation type, which indicates that there is uniform deformation throughout the material [51]. An example of the failure mode observed is shown in Figure 6.

These results agree with the work of Shin et al. [52], who observed that the interlaminar shear strength of carbon-epoxy composites remained constant after long cycles of LEO simulation with UV exposure. Furthermore, it was also observed that the fracture mode of carbon/epoxy and glass/epoxy composites subjected to 6 months of aging with UV exposure did not change compared to the fracture mode of the unaged material [53].

#### 3.6.2. Bending: Impulse Excitation Test

**Figure 7:**
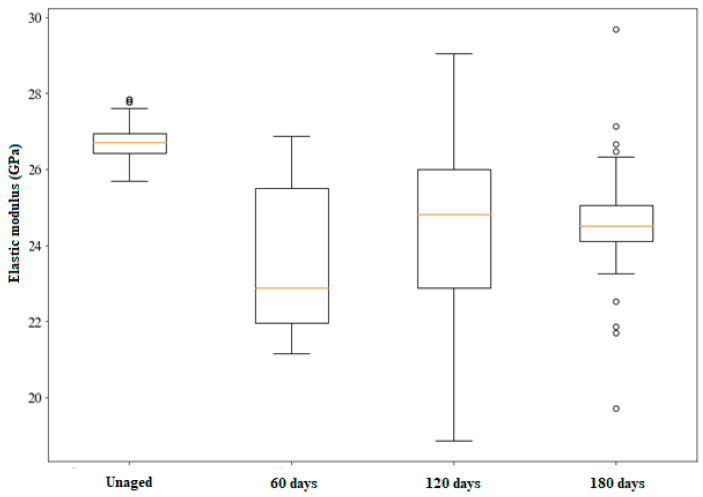
Effect of UV exposure on the elastic modulus. Impulse excitation test. The orange line is the median, and the circles are the outliers.

The results of the non-destructive impulse excitation test comparing the as-manufactured material and the UV-aged but non-sandblasted samples are shown in Table 6. By analyzing the coefficients of variation (CV), it is possible to observe that there is an increase in the dispersion of the values of the aged samples in relation to the non-aged ones. Figure 7 shows the results graphically, where it is possible to clearly see the increase in the dispersion of the values with UV exposure. Due to the high dispersion of the results, it can only be stated that there is a tendency for the elastic modulus value to decrease with aging time, but the values are statistically equal. The increase in dispersion indicates, however, that damage is being generated by the exposure of the material to UV, interfering with the propagation of sound waves [35]. On the other hand, it is observed that the median of the material without aging is higher than the medians of the aged groups as a consequence of the deleterious effect of UV radiation, in agreement with what was reported in previous works [20,46,50,54].

Table 7 presents the mean values, median, and standard deviations for the modulus of elasticity of the samples that were only sandblasted and those with the combined effects of UV aging and sandblasting, as per Table 1. From these results, it is possible to observe a behavior similar to the behavior observed for the composites only subjected to UV aging (Figure 7). This means that there was an increase in the dispersion of the experimental results for all UV aging conditions, with the exception of one; the mean values are statistically equal, and there is a reduction in the median of the aged specimens in relation to the specimens that were only sandblasted. These results seem to indicate that UV exposure, regardless of whether or not it was preceded by sandblasting, was the preponderant effect under the experimental conditions of this work. This result agrees with previous studies, which identified UV exposure as having the most significant effect on the flexural and fatigue strength [55] and impact strength [56] of glass/epoxy composites.

#### 3.6.3. Bending: Three-Point Bending

Table 8 presents the values of the average values and standard deviations of the modulus of elasticity and the flexural rupture stress of the samples without aging and aged by UV as a function of the exposure time. The results obtained are statistically equal, similar to the results obtained for the modulus of elasticity by the impulse excitation test (Table 6).

Two observations can be made regarding the modulus of elasticity when comparing the results in Table 6 and Table 8. First, the values obtained in the flexural test are approximately 20% lower than those obtained in the impulse excitation test. This difference is due to the elastic contribution of the test equipment in measuring the modulus of elasticity when an instrumented test is not performed [57]. Furthermore, although for both tests it can be stated that UV aging did not cause a significant effect on the rigidity of the material, the impulse excitation test showed greater sensitivity, indicating a possible decrease in values as a function of the exposure time. This difference is directly linked to the fact that in the impulse excitation test, any defect generated in the polymer matrix will interfere with the propagation of sound waves, while in the mechanical test, the influence of the matrix on the measurement of mechanical properties is less than that of the fibers. It is important to remember here that UV radiation will initially affect mainly the surface of the material [53]. In other words, there will be a greater effect on the matrix than on the fibers.

The flexural test results of the sandblasted and UV-aged samples are listed in Table 9. The elastic modulus values were similar to those of the samples that were only UV-aged (Table 8). This result indicates that sandblasting did not significantly affect the elastic response of the material, although there was an increase in the dispersion of the results, as observed by the increase in the coefficient of variance values, except for sample #1. This result is directly linked to the fact that both sandblasting and UV-caused defects on the surface of the composite mainly affect the polymer matrix. Since the modulus of elasticity of a composite with glass and/or carbon fibers is a fiber-dominated property, the damage was not sufficient to deteriorate the stiffness of the material. In fact, several studies show that even when there are large delaminations in laminated composites, stiffness is little affected as long as the fibers remain intact or little damaged [22,58].

Regarding flexural strength, there was a reduction in value in relation to the as-manufactured material (Table 8), with the exception of sample #4. It is important to highlight that this sample was the only one in which blasting was performed before UV aging, as listed in Table 1. This behavior seems to indicate that the surface deterioration caused by UV exposure causes a synergistic effect that increases the deterioration of the material when subsequently subjected to sandblasting.

## 4. Conclusions

In this work, the combined effects of UV radiation and particle abrasion on the interlaminar shear strength and flexural behavior of hybrid composites with an epoxy matrix reinforced by glass and carbon fibers were analyzed as a function of the aging time. The results obtained showed that:

- During the analysis period used in this study (180 days), the damage caused by UV radiation in the epoxy matrix was restricted to the outer layers of the composite. Therefore, the interlaminar shear strength, therefore, was not affected by UV radiation since this parameter reflects the shear response of the internal layers of a composite.
- The flexural behavior showed that UV exposure caused a greater effect than sandblasting. Thus, samples that were first exposed to UV and then sandblasted showed the greatest drop in flexural strength. This behavior was attributed to the deterioration of the surface layer by the action of UV, which led to a greater ejection of material from the composite when sandblasted.

From an operational point of view, the results obtained showed that UV protection, either through the use of additives in the polymer matrix or through an external layer of UV-absorbing material, is a key point in maintaining the structural integrity of polymer composites for application in satellites in low Earth orbit and also in exoplanet environments. This protection against UV effects appears to be even more relevant than extra protection against particle abrasion. Of course, it will be necessary to extend the period of exposure to UV rays for longer periods in order to have a better understanding of the effects of long-term degradation in real service situations.

## Figures and Tables

**Table 1:** Sequence of UV aging and abrasion damage.

Samples	Total Aging Time (Days)	Damage Introduction Procedure
#1	60	UV aging for 60 days + sandblasting
#2	120	UV aging for 60 days + sandblasting + aging for 60 days
#3		UV aging for 120 days + sandblasting
#4		sandblasting + UV aging for 120 days
#5	180	UV aging for 60 days + sandblasting + aging for 120 days

**Table 2:** Average values, in mm, of the parameters Rv (peak-to-valley), Rp (peak-to-peak) and Rt (total texture height).

	Rv	Rp	Rt
**Average**	0.335 ± 0.130	1.387 ± 0.392	1.722 ± 0.477

**Table 3:** Colorimetric analysis of the composites before and after aging under UV radiation for 60, 120, and 180 days.

Surface	L*	a*	b*
**As received**	18.876 ± 2.427	−1.274 ± 0.410	4.227 ± 1.271
**UV 60**	19.151 ± 2.795	−1.500 ± 0.602	8.049 ± 1.161
**UV 120**	20.017 ± 1.196	−3.277 ± 0.842	10.515 ± 1.367
**UV 180**	22.955 ± 1.415	−3.215 ± 0.438	9.854 ± 1.718
**Lateral**	L*	a*	b*
**As received**	31.777 ± 8.737	−0.802 ± 0.242	4.420 ± 0.567
**UV 60**	36.064 ± 4.616	−2.543 ± 0.525	12.877 ± 2.892
**UV 120**	31.995 ± 3.369	−2.312 ± 0.353	11.179 ± 1.631
**UV 180**	37.446 ± 2.528	−2.563 ± 0.247	12.422 ± 1.363

**Table 4:** Variation of colorimetric parameters.

**Surface**	***ΔL*** *	***Δa*** ^*^	***Δb*** ^*^	***ΔE*** ^*^
**UV 60**	0.274	−0.226	3.822	3.838
**UV 120**	1.141	−2.003	6.275	6.685
**UV 180**	4.079	−1.941	5.627	7.216
**Side**	ΔL*	Δa*	Δb*	ΔE*
**UV 60**	4.287	−1.743	8.457	9.641
**UV 120**	0.218	−1.509	6.759	6.929
**UV 180**	5.668	−1.761	8.003	9.964

**Table 5:** Mean values and standard deviations of samples after ILSS mechanical test.

	As-Manufactured	60 Days	120 Days	180 Days
**τ (MPa)**	34.3 ± 4.2	28.8 ± 6.1	35.3 ± 6.5	35.7 ± 6.1

**Table 6:** Values of the elasticity modulus obtained through the impulse excitation test. UV-aged samples.

	As-Manufactured	60 Days	120 Days	180 Days
E (GPa)	26.7 ± 0.4	23.5 ± 1.8	24.5 ± 1.7	24.5 ± 1.0
CV (%)	1.6	7.7	6.9	4.0

**Table 7:** Mean and median values of the elastic moduli of the samples only sandblasted (Reference) and of the samples with combined effects of sandblasting and UV.

Samples	E (GPa)	CV (%)	Median (GPa)
Reference	25.7 ± 0.8	2.9	25.7
#1	22.6 ± 3.2	14.0	23.8
#2	24.9 ± 0.1	2.0	24.7
#3	24.3 ± 1.1	4.4	24.2
#4	23.0 ± 2.1	9.3	23.6
#5	23.4 ± 3.3	14.2	23.9

**Table 8:** Average values of the modulus of elasticity (E) and the tensile strength (σr) obtained by three-point bending test. UV-aged samples.

	As-Manufactured	60 Days	120 Days	180 Days
E (GPa)	19.9 ± 2.9	20.9 ± 2.1	20.5 ± 2.4	20.8 ± 1.9
σ_r_ (MPa)	392.5 ± 59.3	379.4 ± 36.3	411.0 ± 44.6	371.6 ± 61.1

**Table 9:** Average values of the modulus of elasticity (E) and the tensile strength (σr) obtained by bending test. Combined effect of sandblasting and UV exposure. The reference samples were only sandblasted.

Samples	E (GPa)	CV (%)	σ_r_ (MPa)	CV (%)
Reference	20.7 ± 1.4	6.6	312.5 ± 42.2	13.5
#1	21.4 ± 1.5	7.0	352.3 ± 65.5	18.6
#2	20.2 ± 2.6	6.6	354.3 ± 44.2	12.5
#3	21.9 ± 1.4	6.3	369.0 ± 15.7	4.2
#4	22.0 ± 2.5	11.4	385.0 ± 45.7	11.9
#5	20.3 ± 3.5	17.1	331.4 ± 38.9	11.7

## Data Availability

Data is contained within the article.
